# It works! Lumpfish can significantly lower sea lice infestation in large-scale salmon farming

**DOI:** 10.1242/bio.036301

**Published:** 2018-09-15

**Authors:** Albert Kjartan Dagbjartarson Imsland, Anna Hanssen, Ane Vigdisdatter Nytrø, Patrick Reynolds, Thor Magne Jonassen, Thor Arne Hangstad, Tor Anders Elvegård, Tonje Cecilie Urskog, Bjørn Mikalsen

**Affiliations:** 1 Akvaplan-niva Iceland Office, Akralind 4, 201 Kópavogur, Iceland; 2 Department of Biology, University of Bergen, High Technology Centre, 5020 Bergen, Norway; 3 Lerøy Aurora, Postbox 2123, 9267 Tromsø, Norway; 4 Akvaplan-niva, Framsenteret, 9296 Tromsø, Norway; 5 Gildeskål Forskningsstasjon (GIFAS) AS, Gildeskål, 8140 Inndyr, Norway; 6 Nordlaks Oppdrett AS, Post box 224, 8455 Stokmarknes, Norway; 7 Grieg Seafood Finnmark AS, Markedsgata 3, Alta, Norway

**Keywords:** Biological delousing, Lumpfish, Sea lice, Atlantic salmon

## Abstract

To assess the efficacy of lumpfish grazing on attached sea lice on Atlantic salmon, six large-scale sea cages, (130 m circumference, 37,688 m^3^ volume) each stocked with approximately 200,000 salmon 0+ smolts, were stocked with a 4, 6 and 8% density (8000, 12,000 and 16,000, respectively) of lumpfish. The sea cages without lumpfish acted as controls. Sea lice infestation levels on the salmon were monitored weekly and bi-weekly from 6 October to 17 May the subsequent year. Mortality of the lumpfish rose with decreasing sea temperatures to around 0.8% week^−1^ and did not vary between the lumpfish groups. There were clear signs of lumpfish grazing on sea lice, with significantly lower average levels of chalimus, pre-adult and adult female *Lepeophtheirus salmonis* and *Caligus elongatus* sea lice per salmon. Lumpfish in the high density (8%) group reduced the mature female *L. salmonis* to levels equal to or lower than the counts recorded prior to the start of the study. Overall, the present results indicate that lumpfish are a suitable cold-water option for biological delousing of Atlantic salmon in large-scale production conditions.

## Abstract (precis)

**Summary:** Lumpfish are effective cleaner fish for biological delousing and can significantly lower average levels of pre-adult and female *Lepeophtheirus salmonis* and *Caligus elongatus* sea lice on Atlantic salmon.

## INTRODUCTION

Global production of Atlantic salmon, *Salmo salar* was 2.07 million tons in 2014, worth over 9 billion Euros (Marine Harvest, 2015), but losses due to sea lice are limiting industry growth and compromising its sustainability (Costello, 2009; Iversen et al., 2015). Like elsewhere in the Atlantic Ocean (Boxaspen, 2006; á Norði et al., 2015), the two most abundant sea lice species on salmon farmed in Norwegian waters are the salmonid specialist, salmon louse *Lepeophtheirus salmonis*, and the teleost generalist *Caligus elongatus*. In the northern parts of Norway, high *C. elongatus* abundance on farmed fish frequently occurs in autumn (Øines et al., 2006). The *L. salmonis* life cycle involves three planktonic larval stages, two non-infective naupliar stages and an infective copepodid stage (Hamre et al., 2013). Water temperature is a key regulator of the development times of all *L. salmonis* stages (Johnson and Albright, 1991; Samsing et al., 2016) and infestation success is greatly reduced at temperatures below 5°C (Samsing et al., 2016). The generation time of salmon lice has been estimated to range between 50 days at 12°C and 114 days at 7°C (Tully, 1992) suggesting that infestations may increase in response to warmer temperatures. *C. elongatus* causes less skin damage to the host (MacKinnon, 1993), but may have farther reaching consequences as they can be carried to, or from, a region on numerous species of wild fish (Kabata, 1979; Øines et al., 2006). The life cycle of *C. elongatus* consists of eight stages: two nauplii, one copepodid, four chalimi, and adult. A separate pre-adult stage does not occur. The copepodid is infective and all subsequent stages live on fish. At 10°C the generation time is 43 days (Piasecki and Mackinnon, 1995). Adult gravid females of *C. elongatus* are common during late autumn and winter (á Norði et al., 2015), hence it has become a usual practise to monitor *C. elongatus* in northern Norway during the winter although Norwegian regulation only requires that *L. salmonis* be monitored on a regular basis (https://lovdata.no/dokument/SF/forskrift/2012-12-05-1140) throughout the production period in the sea.

The biological control of sea lice through the use of ‘cleaner fish’ has recently become a potential alternative due to the increased occurrence of resistant lice, the reduced public acceptance of chemotherapeutic use in food production, and the urgent need for an effective and sustainable method of parasite control in Atlantic salmon aquaculture (Treasurer, 2002; Powell et al., 2018). One major advantage in using cleaner-fish species is that their deployment in sea cages is generally neither stressful for the salmon, nor does it interrupt the host fish's daily routines. In contrast, medicinal treatments usually involve starving the salmon for a period prior to treatment, and after treatment appetite is generally supressed for a period of time. Both factors contribute to lost growth in the salmon.

Lumpfish (or lumpsucker) *Cyclopterus lumpus* L. 1758 is widely distributed across a large area on both sides of the north Atlantic Ocean, from Nunavut, Hudson Bay and Labrador, to New Jersey and Bermuda in the western Atlantic, to the Barents Sea, Iceland and Greenland and the Iberian Peninsula on the eastern side (Vasconcelos et al., 2004; Bañón et al., 2008; Pampoulie et al., 2014). Lumpfish are found all along the Norwegian coastline (Jónsdóttir et al., 2018). Recently the lumpfish has been suggested as a cold-water cleaner fish for removal of sea lice from Atlantic salmon. Initial results in small experimental sea pens (5×5×5 m) were very promising with up to 93–97% less sea lice infection (adult female lice) in sea pens containing lumpfish (Imsland et al., 2014a, b, c, 2015a, b). A recent trial in the Faroes (Eliasen et al., 2018) investigated the stomach content of lumpfish caught from the edge of salmon farming sea pens. They found a clear seasonal effect in lice grazing of lumpfish (most active in the autumn and winter) together with a size effect (smaller lumpfish are more active lice grazers). However, this study did not involve different densities of lumpfish contained in the sea pens, nor did it evaluate lice grazing with and without lumpfish present. Driven by the industry's need for effective sea lice control, commercial production of lumpfish has increased rapidly and reached 15.2 million juveniles in 2015 in Norway (Norwegian Directorate of Fisheries, 2016) and approximately 10 million in the UK, and it is expected to exceed 50 million juveniles in 2018, 40 million in Norway alone (Erlend Waatevik, EWA Consulting, Nodland, personal communication). Up until now, all published data on lumpfish use has come from experiments conducted in small-scale sea pens (Imsland et al., 2014a, b, c, 2015a, b) or in land-based facilities (Nytrø et al., 2014; Imsland et al., 2016a, b, 2018). Consequently, there exists an urgent need to validate the use of lumpfish under commercial production conditions, in large-scale sea pens.

The aim of this study was to quantify the grazing of lumpfish on sea lice by enumerating the different life stages of lice found on Atlantic salmon in large-scale sea cages, with or without lumpfish. The first objective of the current study was to quantify the grazing of sea lice by lumpfish reared under different initial densities in full-scale production conditions. The second objective was to study survival, growth and behaviour of lumpfish during autumn, winter and spring under production conditions in northern Norway. Prior small-scale studies indicate that lumpfish readily graze on *L. salmonis*, but the efficacy may depend on the initial density and size of the lumpfish (Imsland et al., 2014a, 2016a). Whether this also applies to *C. elongatus* is unknown. Also, juvenile lumpfish display good growth between 4 and 16°C (Nytrø et al., 2014), whereas growth and survival below 4°C is unknown. We predicted that lumpfish will be more efficient in sea lice control when reared at greater densities and that lumpfish will readily graze on both sea lice species. Furthermore, we predict that the mortality rate of the lumpfish will increase as sea temperatures decrease below 4°C, approximately.

## RESULTS

### Growth and mortality

**Fig. 1.**
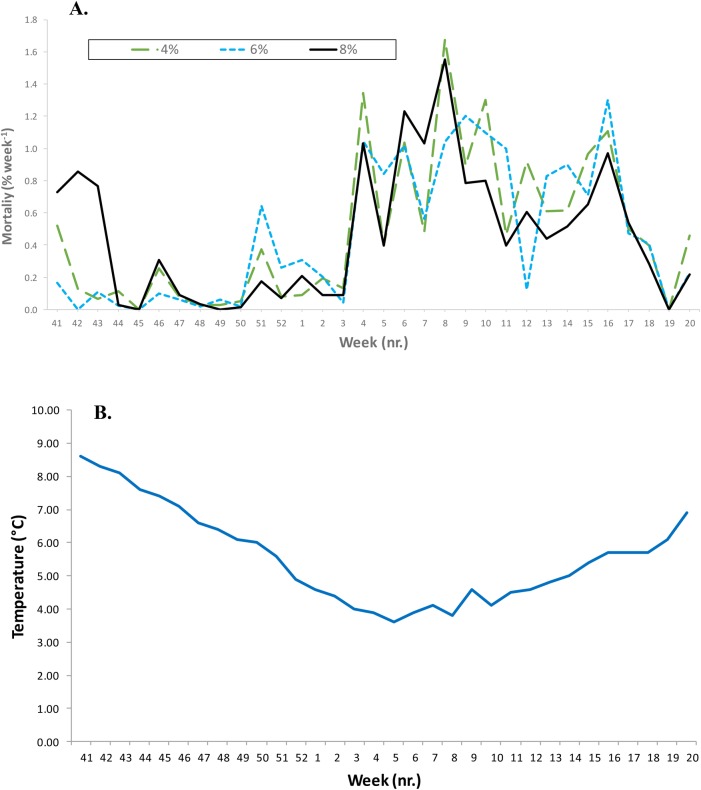
**Mortality and rearing temperature of lumpfish in large-scale sea pens.** Mortality of lumpfish (A) reared in large-scale sea pens with Atlantic salmon from October to May and mean daily temperature (B) at 6 m depth in sea pens during the experimental period.

Final mean (±s.d.) weight of the Atlantic salmon and lumpfish was 712±120 g and 115±27 g, respectively, in May 2016 and did not vary between treatments for either species (two-way nested ANOVA, *P*>0.5). The overall specific growth rate of the lumpfish was 0.68% day^−1^ and did not vary between the density groups (two-way nested ANOVA, *P*>0.6). During the first three weeks of the trial mortality (Fig. 1A) was higher in the 8% group compared to the two other lumpfish groups (χ^2^>12.2, *P*<0.001). Mortality of the lumpfish remained at low levels until early January (Fig. 1A) when it rose in all three lumpfish groups to around 0.8% week^−1^ and did not vary between the lumpfish groups. This increase in mortality coincided with sea temperatures around and below 4°C (Fig. 1B). No antagonistic behaviour between salmon and lumpfish was seen during the whole experimental period.

### Sea lice infestation levels

#### Chalimus stages of *L. salmonis*

**Fig. 2.**
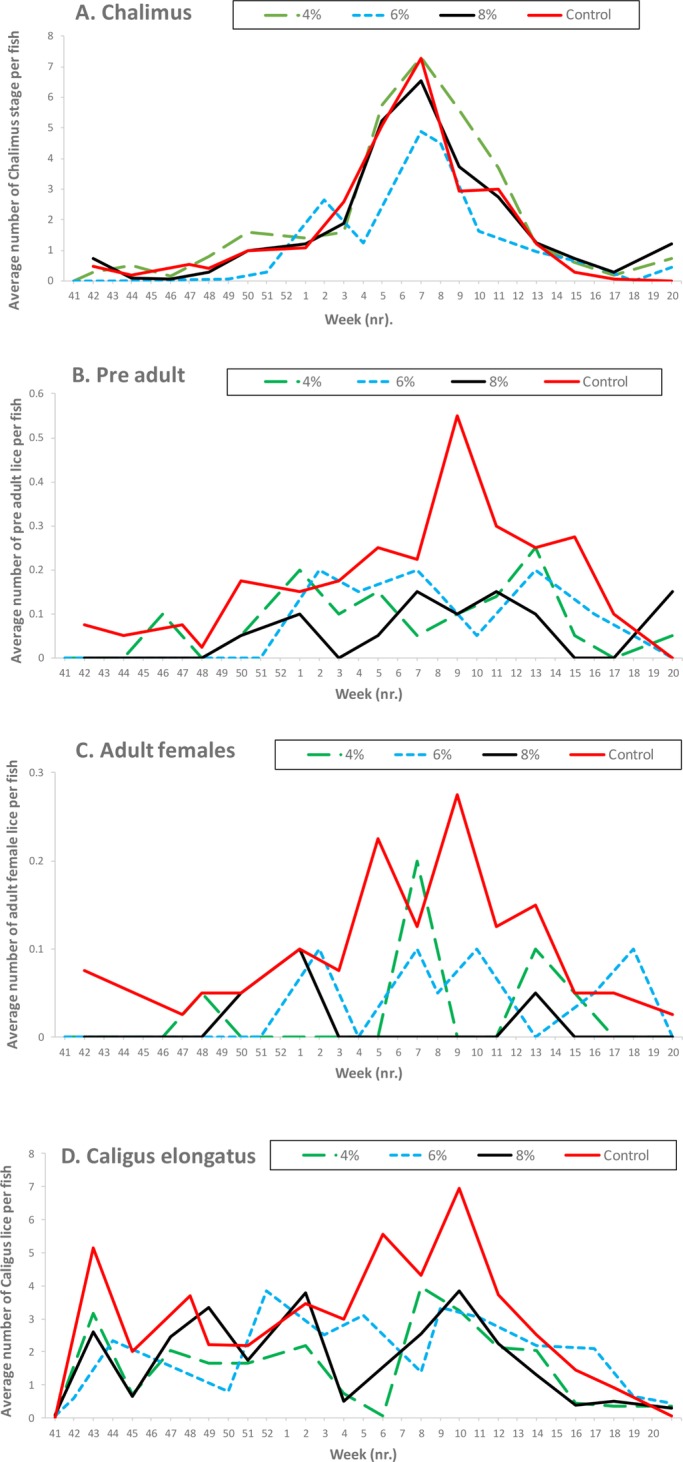
**Assessment of lice grazing of lumpfish in large-scale sea pens.** Occurrence of the chalimus (A), pre-adult (B), adult female (C) *L. salmonis* and (D) *Caligus elongatus* per salmon (*n*=60 in each group at each sampling point) in large-scale sea cages with 0 (control), 4, 6 and 8% density of lumpfish recorded for each duplicate treatment during each of the sampling (bi-weekly) dates.

The pre-treatment count for the chalimus stages was an average 0.47 per fish (Fig. 2A) and remained at low levels in all groups until late January when it rose abruptly in all groups, reaching a peak at five to seven individuals per fish in February. Chalimus was lower in the 6% group compared with the control group in February 2016 (SNK post hoc test, *P*<0.05, Fig. 2A).

#### Pre-adult stages of *L. salmonis*

Levels of pre-adult were low (≤0.25) in all lumpfish groups throughout the experimental period (Fig. 2B). All lumpfish treatment groups had lower levels of pre-adult individuals compared to the control group in February and March (SNK post hoc test, *P*<0.05, Fig. 2B).

#### Mature female *L. salmonis*

The pre-treatment count for mature females in the control group was an average of 0.07 per fish (Fig. 2C), and remained at low levels until January. Significantly fewer mature female lice were seen in the 6 and 8% lumpfish groups compared with the control group, from January to April (SNK post hoc test, *P*<0.05, Fig. 2C). Moreover, there was a relationship between lumpfish density and levels of adult female lice as progressively less adult female lice were seen in the 4% to 8% density groups (Fig. 2C). Between weeks 4–11, 60–100% less adult female lice were seen in the lumpfish groups compared with the control group.

#### *Caligus elongatus* infection levels

Levels of *C. elongatus* rose in all groups during the autumn (Fig. 2D). Significantly lower levels of *C. elongatus* were seen in the lumpfish groups from late February to early April (SNK post hoc test, *P*<0.05, Fig. 2D). In April, the *C. elongatus* levels decreased in all experimental groups and final levels in May were similar to the initial ones in October the year before.

## DISCUSSION

The stage composition of the *L. salmonis* population changed over time with a natural succession from predominantly chalimus, to pre-adult, to adult lice (Fig. 2). During the autumn there were low levels of all lice stages, so the grazing of lumpfish was difficult to evaluate apart from pre-adult levels in the high-density group. As the trial progressed, the evidence became more conclusive; for chalimus stages of *L. salmonis*, it was not until late January and February that there were clear differences between the control group and the 6% lumpfish density group. The size of the lice at this stage of development of *L. salmonis* (1–2 mm) could also be too small to be attractive as a preferred food item for lumpfish, as none were found in the stomachs of the fish when gastric lavage was undertaken in a previous trial (Imsland et al., 2014a).

For pre-adult stages, and particularly in regards to the mature female *L. salmonis*, there were significantly fewer lice attached to Atlantic salmon in cages with lumpfish as compared with the controls. There was clear evidence of grazing from late January onwards. The average numbers of mature female lice remained low throughout the study period for both the 6% and 8% stocked cages, but was more variable in the 4% group. During January to March the average levels of adult female lice per fish had increased to 0.3 per fish in the control group, whereas the 6% and 8% treatments had 73% and 100% fewer mature female lice present compared to the control. This is similar to what has been found under small-scale testing in previous trials (Imsland et al., 2014a, b, 2015a). The low average numbers of mature female lice found in the 6% and 8% lumpfish groups during the latter half of the study period suggest that they are actively selected by lumpfish as a preferred prey item, as suggested by Imsland et al. (2014a). If lumpfish are preferentially selecting the larger mature females, then the potential for re-infestation is significantly diminished.

The average numbers of *C. elongatus* were lower in the groups with lumpfish present, suggesting that lumpfish can be used to reduce the burden of *C. elongatus* in large-scale farming conditions in the sea. This is in line with previous findings in small-scale (125 m^3^ sea pens) rearing conditions (Imsland et al., 2014a).

The overall growth of the lumpfish during the trial was 0.68% day^−1^. This is lower than that found in small-scale trials with similar sized fish (Nytrø et al., 2014), where an average growth of 1.6-1.8% day^−1^ was reported for lumpish reared at constant 4°C, or in ambient water (mean 5.8°C), but comparable to what has been seen in small-scale sea pen trials with similar sized lumpfish (Imsland et al., 2016a). The overall average temperature in the present study was 5.6°C, so present growth was at least 60% lower than can be achieved under controlled conditions in land based tanks (Nytrø et al., 2014). However, the fish in the small-scale trail of Nytrø et al. (2014) were fed *at libitum*, and although the lumpfish were offered feed at the artificial kelp stations, the actual feeding was impossible to monitor in such large-scale conditions. Further, differences in the rearing environment (waves etc.) will likely have a negative effect on the growth potential of the lumpfish when in the sea. However, it must be pointed out that the moderate growth seen in the present study is not negative for the efficiency of the lumpfish for sea lice grazing. An earlier trial pointed out that the sea lice grazing in lumpfish is size dependent (Imsland et al., 2016a) with smaller juveniles (initial size 23 g) consuming on average 30% more sea lice compared to larger (initial size 114 g) juveniles.

No antagonistic behaviour between the two species was observed and the two species seemed to co-exist without issue in the sea pens. Imsland et al. (2014b) pointed out that earlier studies have shown that wild lumpfish and Atlantic salmon post-smolts share feeding grounds in the northeast Atlantic (Holst, 1993; Hansen and Jacobsen, 2000; Jacobsen and Hansen, 2001; Bjelland and Holst, 2004). Similarly, Sheedan et al. (2012) found large numbers of juvenile and adult lumpfish together with Atlantic salmon when sampling Atlantic salmon with surface trawl in the northwest Atlantic (Labrador Sea). The fact that lumpfish and Atlantic salmon share feeding grounds in the wild may help to explain the non-antagonistic behaviour that seemingly has evolved between the two species and can be clearly seen in the present study as well as in previous studies (Imsland et al., 2014a, b, c). Such non-antagonistic behaviour can readily evolve into a form of mutualism as found with parasite cleaning fish species in the tropics (Grutter, 1995; Clague et al., 2011) and between Atlantic salmon and lumpfish during the course of their historical coexistence, as we have suggested in this paper. An example of the opposite was seen in a previous trial with Atlantic cod, *Gadus morhua* (Imsland et al., unpublished). Juvenile lumpfish were added in sea pens with Atlantic cod infected with *C. elongatus* sea lice, and clear signs of antagonistic behaviour were seen where the cod directly attached the lumpfish instead of allowing for cleaning of sea lice from their skin.

No differences in growth between salmon farmed in duoculture with and without lumpfish was seen in present study. Recent trials of duoculture of salmon and cleaner fish indicate little or no effect of cleaner fish on growth performance of salmon (Imsland et al., 2014a). A similar finding has been seen for wrasses; Skiftesvik et al. (2013) concluded that the presence of ballan wrasse (*Labrus bergylta*) did not affect the growth of salmon. In contrast, Imsland et al. (2014c) found indications that cleaner fish can, to a certain degree, have an effect on growth performance of Atlantic salmon in sea pens, but that those effects are minor.

### Conclusions

Sea lice of both species were actively grazed upon, resulting in lower average numbers per fish of chalimus, pre adult and mature female *L. salmonis* when lumpfish were present in the cages. This is in line with our initial prediction for the study. Lumpfish in the high density group (8%) supressed the numbers of mature female *L. salmonis* to levels equal to or lower than the pre-treatment count. Lumpfish mortality increased from late January, which coincided with sea temperatures around and below 4°C.

## MATERIALS AND METHODS

### Atlantic salmon

The salmon used in the study were 0+ smolts produced at the commercial smolt hatchery Lerøy Aurora (Laksefjord, Finnmark, Norway) and were moved to sea cages in August 2015. The salmon were from the Aqua Gen strain and were vaccinated with Pentium Forte Plus (Novartis Aqua, Oslo, Norway).

### Lumpfish

The lumpfish were produced from fertilised eggs from Senja Akvakultursenter AS, Tromsø. The eggs were incubated at 10–12°C and the juveniles were initially fed with Gemma Micro (150–500 μm, Skretting, Norway). After 30 days, the juveniles were fed with 300–1200 μm dry feed pellets (Gemma Wean Diamond, Skretting, Norway). The fish were vaccinated with ALPHA JECT Marin micro 5 (Pharmaq AS, Oslo, Norway) around 500 d° prior to transport to the experimental facility.

### Experimental design

A large-scale experiment was performed at a commercial Atlantic salmon sea farm at 69.80°N, 19.41°E (Lerøy Aurora, Troms county, Norway) from 6 October 2015 to 17 May 2016. The experiment was conducted in eight large sea cages (130 m circumference, 37,688 m^3^ volume) holding 0+ smolts of Atlantic salmon (*n*=193,304±2089 fish pen^−1^) with an initial mean (±s.e.m.) body weight of 198±20 g. On 6 October six cages were stocked with juvenile lumpfish at 4, 6 and 8% density (8000, 12,000 and 16,000, respectively) with a mean (±s.e.m.) body size of 25±2 g. Mortality of the lumpfish was monitored throughout the trial period. During the winter (December to March) each sea pen was offered additional lighting using four 360 W Blue LED light per sea pen (AKVA group ASA, Tromsø, Norway). Water temperature at 6 m depth ranged between 8.3°C in November to 3.6°C in March to 6.8°C in May 2016 (Fig. 1B). The salmon were fed a commercial diet according to the manufacturer’s recommendations (Biomar, Myre, Norway). In each sea pen there were two small (4 m circumference, 10 m deep) plastic kelp stations used as substrate and shelter for the lumpfish. The lumpfish were offered additional feed at 2% BW d^−1^ (Skretting, Norway) at or near the artificial kelp stations, dispersed at 1 m below the water surface.

The experiment described was approved by the local responsible laboratory animal science specialist under the surveillance of the Norwegian Animal Research Authority (NARA) and registered by the authority.

### Growth and performance

The Atlantic salmon and lumpfish were bulk weighed at the start and termination of the trial. Weighing was undertaken without prior starvation. Specific growth rate (SGR) of the lumpfish was calculated according to the formula of Houde and Schekter (1981):

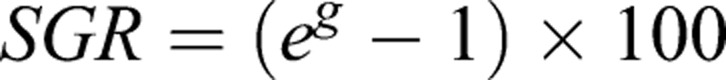
where g=(ln (W_2_)-ln (W_1_)/(t_2_-t_1_) and W_2_ and W_1_ are weights on days t_2_ and t_1_, respectively.

### Assessment of sea lice infestation levels

At the start of the trial, a lice count was undertaken as the fish were transferred into the trial cages. 30 fish were sedated, individually weighed and any lice present were recorded. After the counting was complete, any lice remaining in the container were also recorded. Lice were registered in four categories: (1) *Lepeophtheirus salmonis*, adult female; (2) *L. salmonis*, pre-adult; (3) *L. salmonis*, chalimus; (4) *Caligus elongates*.

According to Norwegian regulation (https://lovdata.no/dokument/SF/forskrift/2012-12-05-1140) sea lice were counted every week when the sea temperature was above 4°C, but every second week when it was under 4°C. The regulation requires that the amount of adult female *L. salmonis* must be below 0.5 lice per salmon in the period from week 27 (July) to week 21 (May). During every lice count, 30 salmon per cage (N_total_=240) were sedated and utilised for counting lice using the same classification as used at the start of the trial. Sea lice infestations on all experimental stocks were natural populations and not experimental introductions.

### Statistics

All statistical analyses were conducted using TIBCO Statistica™ 13.3 software (https://docs.tibco.com/products/tibco-statistica-13-3-0). A Kolmogorov–Smirnov test (Zar, 1984) was used to assess for normality of distributions. The homogeneity of variances was tested using Levene's F test (Zar, 1984). Possible differences in salmon and lumpfish mean weights, lumpfish growth and sea lice infections among treatments were tested with two-way nested analysis of variance (ANOVA), where replicates were nested within treatments. Significant ANOVA were followed by a Student–Newman–Keuls (SNK) multiple comparison test (Zar, 1984) to identify differences among treatments. Data on mortality was tested with a χ^2^ test with the mortality in the lowest density group (4%) used as the expected value. A significance level (α) of 0.05 was used if not stated otherwise.
